# Crystallin β-b2 promotes retinal ganglion cell protection in experimental autoimmune uveoretinitis

**DOI:** 10.3389/fncel.2024.1379540

**Published:** 2024-09-10

**Authors:** Dirk Bauer, Michael R. R. Böhm, Xiaoyu Wu, Bo Wang, Tida Viola Jalilvand, Martin Busch, Maren Kasper, Katrin Brockhaus, Lena Wildschütz, Harutyun Melkonyan, Björn Laffer, Gerd Meyer Zu Hörste, Arnd Heiligenhaus, Solon Thanos

**Affiliations:** ^1^Department of Ophthalmology and Ophtha-Lab at St. Franziskus Hospital, Münster, Germany; ^2^Institute for Experimental Ophthalmology, Westfalian-Wilhelms-University of Münster, Münster, Germany; ^3^Department of Ophthalmology, University of Duisburg-Essen, Essen, Germany; ^4^Institute for Physiological Biochemistry, Westfalian-Wilhelms-University of Münster, Münster, Germany; ^5^Department of Neurology, University Hospital Münster, Münster, Germany

**Keywords:** crystallin β-b2, experimental autoimmune uveitis, neuroprotection, retinal ganglion cell, apoptosis

## Abstract

Crystallin βb2 (crybb2) is upregulated in regenerating retinas and in various pathological conditions of the retina, including uveoretinitis. However, the role of crybb2 in this disease is largely unknown. Therefore, we used recombinant crybb2 (rcrybb2) as intravitreal treatment of B10.RIII mice prior to immunization with human interphotoreceptor retinoid-binding protein peptide 161–180 (hIRBPp161-180) in complete Freund’s adjuvant (CFA) and concomitant injection of pertussis toxin (PTX) to induce experimental autoimmune uveoretinitis (EAU). In naïve mice, more beta III-tubulin (TUBB3) + and RNA-binding protein with multiple splicing (RBPMS) + cells were found in the ganglion cell layer of the retina than in EAU eyes, suggesting a loss of retinal ganglion cells (RGC) during the development of EAU. At the same time, the number of glial fibrillary acidic protein (GFAP) + cells increased in EAU eyes. RGCs were better protected in EAU eyes treated with rcrybb2, while the number of GFAP+ cells decreased. However, in retinal flatmounts, both retinal ganglion cells and retinal endothelial cells stained positive for TUBB3, indicating that TUBB3 is present in naïve B10.RIII mouse eyes not exclusive to RGCs. A significant decline in the number of RBPMS-positive retinal ganglion cells was observed in retinal flatmounts from EAU retinas in comparison to naïve retinas or EAU retinas with intravitreal rcrybb2 treatment. Whereas no significant decrease in TUBB3 levels was detected using Western blot and RT-qPCR, GFAP level, as a marker for astrocytes, increased in EAU mice compared to naïve mice. Level of *Bax* and *Bcl2* in the retina was altered by treatment, suggesting better cell survival and inhibition of apoptosis. Furthermore, our histologic observations of the eyes showed no change in the incidence and severity of EAU, nor was the immune response affected by intravitreal rcrybb2 treatment. Taken together, these results suggest that intravitreal injection of rcrybb2 reduces retinal RGC death during the course of EAU, independent of local or systemic autoimmune responses. In the future, treating posterior uveitis with rcrybb2 to protect RGCs may offer a promising novel therapeutic strategy.

## Introduction

Crystallin α, β, and γ have all been shown to have protective functions for retinal ganglion cells (RGCs). Expression of crystallin increases in the inner segments of photoreceptor neurons in retinas affected by oxidative damage, suggesting that it is involved in RGC axonal regeneration (Bohm et al., 2013; Lee et al., 2011; Li et al., 2021; Thanos et al., 2014).

The survival rate of RGCs generally improves when these cells are exposed to factors released after lens injury (Fischer et al., 2008; Fischer et al., 2004; Sinha et al., 1998). Crystallins, including crystallin βb2 (crybb2), are expressed within the retina, also in filopodial protrusions and axons of RGCs. Indeed, crystallin expression was stronger in the RGC layer (Piri et al., 2007; Thanos et al., 2012). Crybb2 overexpression in RGCs stimulates axon growth in primary hippocampal neurons and retinal explants. Crybb2-transfected cultures also produce conditioned medium, which facilitates axon growth (Liedtke et al., 2007).

Previous studies have shown that crybb2 has a strong neuroprotective and regenerative potential in RGCs, similar to the effect of lens injury (Fischer et al., 2008). However, axon regeneration is a multifactorial process involving several factors in addition to crystallin (Liedtke et al., 2007).

Neuronal crystallins have been found to be suitable biomarkers for monitoring the progression of neuropathy and neuroprotective effects in rat models of ocular hypertension (Prokosch et al., 2013). In a subsequent study, the neuroprotective properties of crystalline CRYAB, CRYBB2, and CRYGB were demonstrated in retinal cultures. Müller cells took up the added crystallines, resulting in increased secretion of various neurotrophic factors in the supernatant, including nerve growth factor, clusterin, and MMP-9 (Liu et al., 2022).

Functionally, β-crystallin has been implicated in protecting the retina from intense light exposure. Two members of the β-crystallin family, crybb1 and crybb2, were identified in drusen preparations isolated from the retina of donor eyes with age-related macular degeneration (AMD), the leading cause of blindness in the elderly population of developed countries. Although variant alleles of the crybb1 and crybb2 genes have been found, none are considered pathogenic (Sturgill et al., 2010).

Mutations in the crybb2 gene can lead to congenital cataracts, and an association of the gene with cataract was previously reported (Ching et al., 2019; Xu et al., 2021a; Zhang et al., 2008). In humans, there is a second βb2 -crystallin derived pseudogene (CRYBB2P1). Conversion of the βb2-crystallin locus to the pseudogene results in lens opacity and cataract (Vanita Sarhadi et al., 2001; Xu et al., 2021b; Zhuang et al., 2019).

Previous studies have reported upregulation of CRYBB2 expression in various ocular pathologies, including age-related macular degeneration (Johnson et al., 2005; Umeda et al., 2005), glaucomatous neuropathy (Prokosch et al., 2013), cauterization-induced ocular hypertension in rats (Prokosch et al., 2013), and ocular hypertension in rats (Chiu et al., 2010; Johnson et al., 2005).

A meta-analysis has revealed a strong association between CRYBB2 expression in the human cortex and certain mental illnesses, including attention-deficit hyperactivity disorder, autism, major depressive disorder, bipolar disorder, and schizophrenia (Graw, 2017; Kim et al., 2014).

Recent research in tumor research indicates that CRYBB2 is involved in carcinogenesis. The differential expression of CRYBB2 and the pseudogene CRYBB2P1 contributes to poor outcomes of breast cancer in African American women by affecting tumor cell proliferation, invasion, metastasis, and tumor immunity (Field et al., 2012). However, a recent analysis identified CRYBB2 as one of 13 genes significantly associated with increased survival in African American glioma patients compared to Caucasian American glioma patients (Wu et al., 2019).

Autoimmune posterior uveitis in humans is associated with chronic or recurrent inflammatory episodes that eventually significantly impair vision. Experimental autoimmune uveoretinitis (EAU) in mice is an eye-specific, T cell-dependent inflammatory response that models certain aspects of posterior uveitis in humans (Caspi, 2011; Caspi et al., 1990). Autoimmune processes that cause uveoretinitis can promote degeneration of the neuronal photoreceptors in the retina, ultimately leading to blindness (Nussenblatt, 2002; Saraswathy et al., 2010). Oxidative stress induced by innate immune processes mediated by macrophages from the blood; (Forrester et al., 1998; Rao, 1990) can mediate photoreceptor degeneration in EAU by activating toll-like receptors and via adaptive immune responses mediated by T-cells in the retina (Caspi, 2003; Rao et al., 2012).

However, previous studies have shown that oxidative stress, peroxynitrate-mediated nitration of photoreceptor mitochondrial proteins and cytochrome c, also develops in the early stages of EAU, even before leukocytes have infiltrated the retina, suggesting that such early responses may constitute important initial pathologic effector events in EAU-related photoreceptor damage (Saraswathy et al., 2010).

Previously, studies showed that early expression of αA-crystallin is associated with the protection of photoreceptor neurons in EAU (Nguyen and Rao, 2010; Rao et al., 2008). In a subsequent publication, Rao et al. (2012) demonstrated that EAU scores were lower in αA-crystallin KO mice following systemic daily administration of αA-crystallin protein beginning on day 12 post-immunization (p.i.). They also found reduced cytokine responses in the retina and increased levels of the anti-inflammatory cytokine interleukin (IL)-10 (Rao et al., 2012).

A previous study found that αA- and βB2-crystallins are not only present in the cytoplasm of retinal cells, but that they are also expressed in the mitochondria during the early stages of EAU and that this is associated with the prevention of retinal cell death (Saraswathy and Rao, 2009). While several studies have demonstrated the role of αA-crystallin in EAU (Rao et al., 2008), little information is available on the role of the β/γ-crystallins in progression of EAU, or as to whether crybb2 potentially protects the retina during the development of EAU. Using the EAU mouse model, we have now elucidated the potential influence of intravitreally injected rcrybb2 on the immune responses that influence EAU pathology and investigated its impact on retinal architecture.

## Materials and methods

### Mice

Male B10.RIII mice (8–12 weeks of age) were purchased from Charles River (Sulzfeld, Germany) and housed under an inverted 12:12-h light–dark schedule. Water and food were available *ad libitum*. Animal experiments conformed with the German regulations of the Society for Laboratory Animal Science (GV-SOLAS) and the European Health Law of the Federation of Laboratory Animal Science Associations (FELASA). The protocol (84–02.04.2015.A252) was approved by the North Rhine-Westphalia State Agency for Nature, Environment, and Consumer Protection (LANUV). All experimental procedures were conducted in accordance with the Institutional Animal Care and Use Committee and with the Association for Research in Vision and Ophthalmology resolution on the use of animals in research. Treatments of mice eyes were performed under general anesthesia, using a ketamine (2 mg/kg per body weight; Ceva-Sanofi, Duesseldorf, Germany) and xylazine (1 mg/kg per body weight; Ceva-Sanofi) injected intraperitoneally.

### Preparation of recombinant crybb2

For preparing recombinant crybb2 (rcrybb2), crybb2-cDNA was inserted into the pQE32 pLasmid (Quiagen, Hilden, Germany) and cloned using a bacterial culture. The bacterial culture was centrifuged (1,500 x *g*) and resuspended in buffer 1 (8 M urea, 50 mM NaH_2_PO_4_, 15 mM imidazole, 10 mM Tris–HCl, and 100 mM NaCl; pH 8.0), all purchased from Sigma-Aldrich (Taufkirchen, Germany). After cloning, the cell lysate was centrifuged at 20,000 x *g* for 30 min. The supernatants were collected and purified using immobilized metal affinity chromatography (Takara Clontech, France). Elution was performed using buffer 1 at pH 6.0, and 100 mM imidazole and the eluate were then dialyzed in distilled water. The identity and purity of βb2-crystallin was verified by sodium dodecyl sulfate (SDS)-polyacrylamide gel electrophoresis (Sigma-Aldrich) and Western blotting. The synthesized βb2-crystallin was dissolved in phosphate-buffered saline (PBS; Sigma-Aldrich) for intravitreal injections (Bohm et al., 2013; Bohm et al., 2015).

### Injections into the eye

For local treatment, the mice received an intravitreal injection of 2 μL of PBS or 2 μL [3 μg/2 μL] rcrybb2 unilaterally (left eye) through the sclera using a glass capillary 3 days prior EAU induction. The lens was protected from damage, as it contains numerous crystalline structures that could distort the experiments. In some control animals, a lens injury was deliberately inflicted, and an additional 2 μl of PBS was injected into the lens to augment the impact of the lens injury. In a separate control group, animals were not subjected to any treatment (Bohm et al., 2012).

### EAU induction and EAU scoring

To induce EAU, B10.RIII mice were treated with hIRBPp161-180 (SGIPYIISYLHPGNTILHVD, EMC Microcollection, Tuebingen, Germany; 100 μg per mouse) emulsified in CFA 1:1 vol/vol (Sigma-Aldrich). Then, 200 μL of the emulsion was injected subcutaneously into each thigh and at the base of the tail. Additionally, 0.4 μg pertussis toxin (PTX; Sigma-Aldrich) in 100 μL PBS was injected intraperitoneally (Hennig et al., 2012). EAU scores were assessed on day 21 p.i. after the mice were sacrificed (Caspi, 2002).

### Histology and immunofluorescence microscopy

For histology, eyes were enucleated and fixed in 80% isopropanol, 25% acetic acid, and 37% formaldehyde (8:1:1; v/v). Eyes were then dehydrated in isopropanol and embedded in paraffin. Mediosagittal sections (7 μm) were prepared by using a rotary microtome (Leica, Wetzlar, Germany) and stained with hematoxylin–eosin. At least three representative sections per eye (*n* = 20 each group) were used for grading the EAU score in a masked fashion. The EAU score was dependent on the number, type, and size of the lesions (Agarwal and Caspi, 2004; Busch et al., 2013; Hennig et al., 2012; Kasper et al., 2018a; Kasper et al., 2018b).

For immunofluorescence staining, the sections were deparaffinized in xylene, hydrated in ethanol (100, 95 and 80%) and then rinsed in distilled water. Subsequently, heat-induced antigen retrieval was conducted using sodium citrate buffer (10 mM sodium citrate, 0.05% Tween, pH 6.0, heated to 95°C for 30 min).The sections were then incubated for 24 h with the primary antibody targeting beta III-tubulin (TUBB3, MA1-118X, Thermo Fisher, Germany), RNA-binding protein with multiple splicing (RBPMS, GTX118619, Genetex, CA, United States) glial fibrillary acidic protein (GFAP, DakoCytomation, Hamburg, Germany; 1:400 in 10% FCS) or polyclonal rabbit anti-crybb2. After rinsing, the slides were incubated (1:200 in 10% FCS) for 30 min at room temperature, (RT) with the secondary antibody (anti-rabbit Cy2 antibody; Dianova, Hamburg, Germany).

Cell nuclei were stained with 4`,6-diamino-2-phenylindole dihydrochloride hydrate (working concentration: 1 μg/mL; DAPI; Sigma-Aldrich). The slides were then coverslipped with Mowiol (Carl Roth, Karlsruhe, Germany). Sections were examined under a fluorescence microscope (Axiophot, Carl Zeiss, Oberkochen, Germany). At least three representative sections per eye (*N* = 5 per group) were used for localization of positive cells (three randomized regions per eye) within a region of 0.15 mm^2^. Sections without primary antibodies were used as negative controls (Bohm et al., 2012; Prokosch et al., 2013). The retinas (N = 4 per group) were initially fixed for a period of 4 h with 4% PFA in 1x PBS at a temperature of 4–8°C. Thereafter, the retinal flat mounts were treated with 30% sucrose in 1xPBS at 4°C for 24 h and thereafter blocked with goat serum for 1 h at room. The flat mounts were stained overnight with a polyclonal rabbit anti-RBPMS antibody or a monoclonal rabbit anti-CD31/PECAM1 and monoclonal mouse anti-TUBB3 (RBPMS: 1:200 dilution, GTX118619, CD31/PECAM1: 1:200 dilution, A19014, Abclonal, Wuhan, China, TUBB3: 1:200 dilution, MA1-118X, Thermo Fisher, Germany) in PBST-X (0.3% Triton X-100 in PBS). Subsequently, the flat mounts were incubated for 1 h with goat anti-mouse AlexaFluor™ 488 (1:1000 dilution, M30001, Thermo Fisher, Germany) and goat anti-rabbit AlexaFluor™ 594 (1:1000 dilution, A11012, Thermo Fisher). Following three washes in PBST-X, the retinas were meticulously prepared on diagnostic microscope slides (Thermo Scientific). Subsequently, the flat mounts were embedded with Mowiol (Carl Roth). The sections were examined under a fluorescence microscope (Axiophot, Carl Zeiss, Oberkochen, Germany) and three randomly selected regions per retina were photographed. The number of RBPMS-positive cells in each region was enumerated in a masked fashion, and the means were calculated for utilization in the statistical analysis.

### Western blot

The Western blotting test was performed using the eyes of four animals per group, which were pooled together. Extraocular tissues were removed. The retina was then carefully separated and immediately shock-frozen in liquid nitrogen. Tissues were stored at −80°C until use. For Western blotting, the tissues were homogenized in SDS sample buffer containing 130 mM Tris–HCl (Roth, Karlsruhe, Germany), 10% w/v SDS, 10% mercaptophenol, 20% glycerol, and 0.06% w/v bromophenol blue (all purchased from Sigma-Aldrich). Samples were sonicated and heated, and the protein concentration was calculated by using the Bradford reagents (Bio-Rad, Hercules, CA, United States). Then, 50 μg of proteins were loaded onto SDS-polyacrylamide gels using a protein size ladder (Bio-Rad). Proteins were blotted onto a nitrocellulose membrane (Whatman, GE Healthcare Europe GmbH, Freiburg, Germany). Blots were blocked for 1 h in 5% (w/v) fat-free dry milk (Carl Roth, Karlsruhe, Germany) in PBS with 0.1% (v/v) Tween-20 (PBS-T). The blots were then incubated overnight at 4°C with polyclonal anti-rabbit TUBB3 (1:500), GFAP (1:500), or glyceraldehyde 3-phosphate dehydrogenase (GAPDH, 1:10000; Santa Cruz Biotechnology, Santa Cruz, CA, United States). The blot membrane was then incubated with the horseradish peroxidase-conjugated secondary antibody (Sigma-Aldrich) in blocking solution for 1 h at RT. Positive binding was detected by enhanced chemiluminescence (Amersham, Rockville, United Kingdom). The relative densities of proteins were determined using Alpha EaseFC (Alpha-Ease FC software 4.0, Alpha Innotech, Biozym Scientific, Vienna, Austria; Bohm et al., 2015; Bohm et al., 2016).

After subtracting the specific background density in the surrounding region, the protein density of a fixed area was determined for each spot. The density of the spots was correlated and corrected for the relative density of the respective application control. The spot density of the control group samples was defined as the respective reference value and the relative values of the other groups were calculated. The blots were repeated once, and the relative density was averaged.

### RT-qPCR

Total RNA was isolated from pooled retinas [naïve mice *n* = 5; EAU (untreated) *n* = 5, EAU + PBS (vitreous) *n* = 5; EAU + rcrybb2 (vitreous) *n* = 5]. RNA was isolated using Gene Elute Mammalian Total RNA Miniprep Kit (Sigma-Aldrich) according to the manufacturer’s protocol. RNA was quantified using a UV/visual spectral photometer (NanoDrop ND-1000, Peqlab, Erlangen, Germany). The High Capacity cDNA Reverse Transcription Kit (ABI, Foster City, CA, United States) was used to reverse transcribe 1 μg of total RNA into complementary DNA (cDNA). The RT-qPCR primer pairs designed for SYBR-Green-based RT-qPCR were used for the analysis:

*Bcl-2* (NM_009741.3): forward, 5´-GCCCCAGCATGCGACCTCTG-3′; reverse, 5´-AGTGATGCAGGCCCCGACCA-3′.

Bcl-2-associated X-protein (*Bax*; NM_007527): forward, 5´-GCTGAGCGAGTGTCTCCGGC-3′; reverse, 5´-GGGGAGTCCGTGTCCACGTCA-3′.

beta III-tubulin (*Tubb3*; NM 22152): forward, 5′- CATCAGCGATGAGCACGGCATA-3′; reverse, 5`-GGTTCCAAGTCCACCAGAATGG-3′.

Gfap (NM_14580): forward, 5′- CACCTACAGGAAATTGCTGGAGG −3′; reverse, 5′- CCACGATGTTCCTCTTGAGGTG-3´.

Real-time PCR was performed in triplicate, using a SYBR-PCR Kit, according to the protocol provided by the manufacturer (Applied Biosystems). The data were analyzed using SDS 2.2 software (Applied Biosystems). Relative expression was calculated as $2-\Delta Ct\left(\mathrm{specific}\;\mathrm{gene}\right)/2-\Delta Ct\;\mathrm{mean}$, using the gene encoding Gapdh as an endogenous housekeeping control gene. Relative expression (RQ, relative quotient) was calculated and expressed as fold change relative to the expression level in the control group (Bohm et al., 2016).

### Cell culture

Splenocytes from EAU mice were isolated from the spleen, triturated, and treated with hemolysis buffer (155 mM NH_4_Cl, 126.6 mM EDTA, 9.9 mM NaHCO_3_; pH 7.3; Biochrom, Berlin, Germany). After hemolysis, the cells were washed and then suspended at a concentration of 5×10^6^ cells/ml in lymphocyte medium in a sterile 24-well plate. To activate the splenocytes, 10 μg/mL hIRBPp161-180 peptide, 5 μg/mL concanavalin A (ConA, Biochrom), mAb targeting CD3e (1 μL/mL), or medium (control) was added. After 24 h, the supernatants were harvested and stored at −80°C until use for enzyme-linked immunosorbent assay (ELISA; Bauer et al., 2009; Hennig et al., 2012).

### Quantification of cytokines via ELISA

The level of various cytokines, including interferon (IFN)-γ, IL-6, IL-10, and IL-17, in cell culture supernatants were examined by using commercially available ELISA kits [OptEIA (PharMingen, Hamburg, Germany); IL-17 Duoset (R&D Systems GmbH, Wiesbaden- Nordenstadt, Germany); Bauer et al., 2017].

### Single-cell RNA-sequencing analysis of retinal endothelial cells during experimental autoimmune uveitis

Single-cell RNA-sequencing (scRNA-seq) data from a previous study by Lipski et al. (2020) of sorted retinal endothelial cells from a pool of four healthy mice and three mice with EAU (EAU, d21, C57/Bl6 wild-type mice were used for the study) were obtained and downloaded from the Gene Expression Omnibus (GEO) repository with the accession number GSE144168. The normalized data were processed by using the NetworkAnalyst 3.0 platform^1^ (Zhou et al., 2019). Differential analysis between two groups (EAU group vs. naïve group) was performed using the “Limma” R package in the online platform. The classical Bayesian algorithm was used to calculate the differentially expressed genes in the GSE144168 dataset. The absolute value of Log2Fold > 1.0 and adj. *p* < 0.05 were used as significance indicators.

### Statistical analysis

Student’s *t*-test (comparison of two groups) or one-way ANOVA (comparison of three or more groups) was used to analyze normally distributed data. Welch-test was used when homogeneity of variance was not adequate. For EAU scoring data, a nonparametric test was used (U-Test for comparison of two groups; Kruskal-Wallis-test for comparison of three or more groups). Relative protein densities in Western blots are shown as mean ± SEM values. *p* ≤ 0.05 was considered as statistically significant.

## Results

### Intraocular localization of intravitreally injected rcrybb2

**Figure 1:**
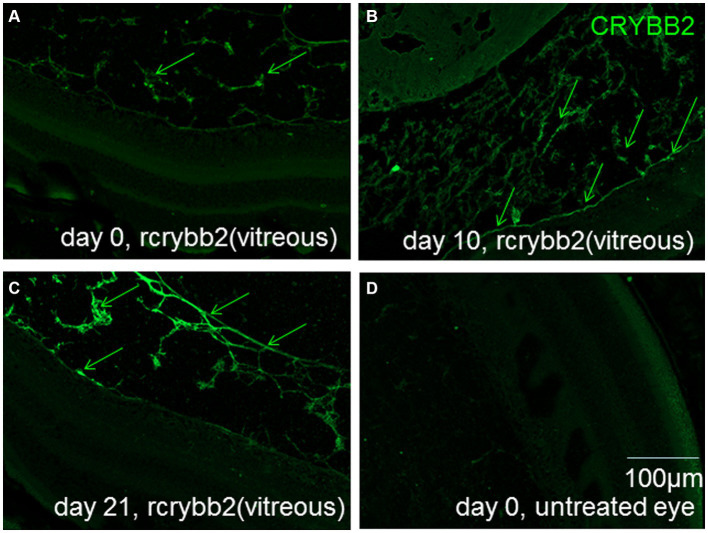
Localization of rcrybb2 in the eyes after injection into naïve mice. Paraffin-embedded sections of eyes (*N* = 3 at each time point) after injection of 3 μg/2 μL rcrybb2 and immunofluorescence staining with an antibody directed against crybb2 showing pronounced fluorescent staining. Paraffin-embedded sections of mouse eyes at 0 **(A)**, 10 **(B)**, and 21 days **(C)** after injection of 3 μg/2 μL rcrybb2 showing that rcrybb2 could still be detected after 21 days. **(D)** Paraffin-embedded section of an untreated mouse eye. Scale bar: 100 μm.

Experiments were performed to determine the presence and distribution of rcrybb2 in the mouse eye after intravitreal injection of 3 μg / 2 μL of rcrybb2 into the vitreous of the left eye of naïve mice. Control groups of mice were left untreated. After a single intravitreal injection of rcrybb2 [rcrybb2(vitreous)], pronounced immunofluorescent staining of the vitreous, vitreoretinal interface, and ganglion cell layer was detected by fluorescence microscopy using a polyclonal antibody directed against crybb2, and this was also detectable 10 or 21 days after injection (Figure 1).

### Expression of TUBB3, RBPMS, and GFAP in EAU retinas after intravitreal injection of rcrybb2

**Figure 2:**
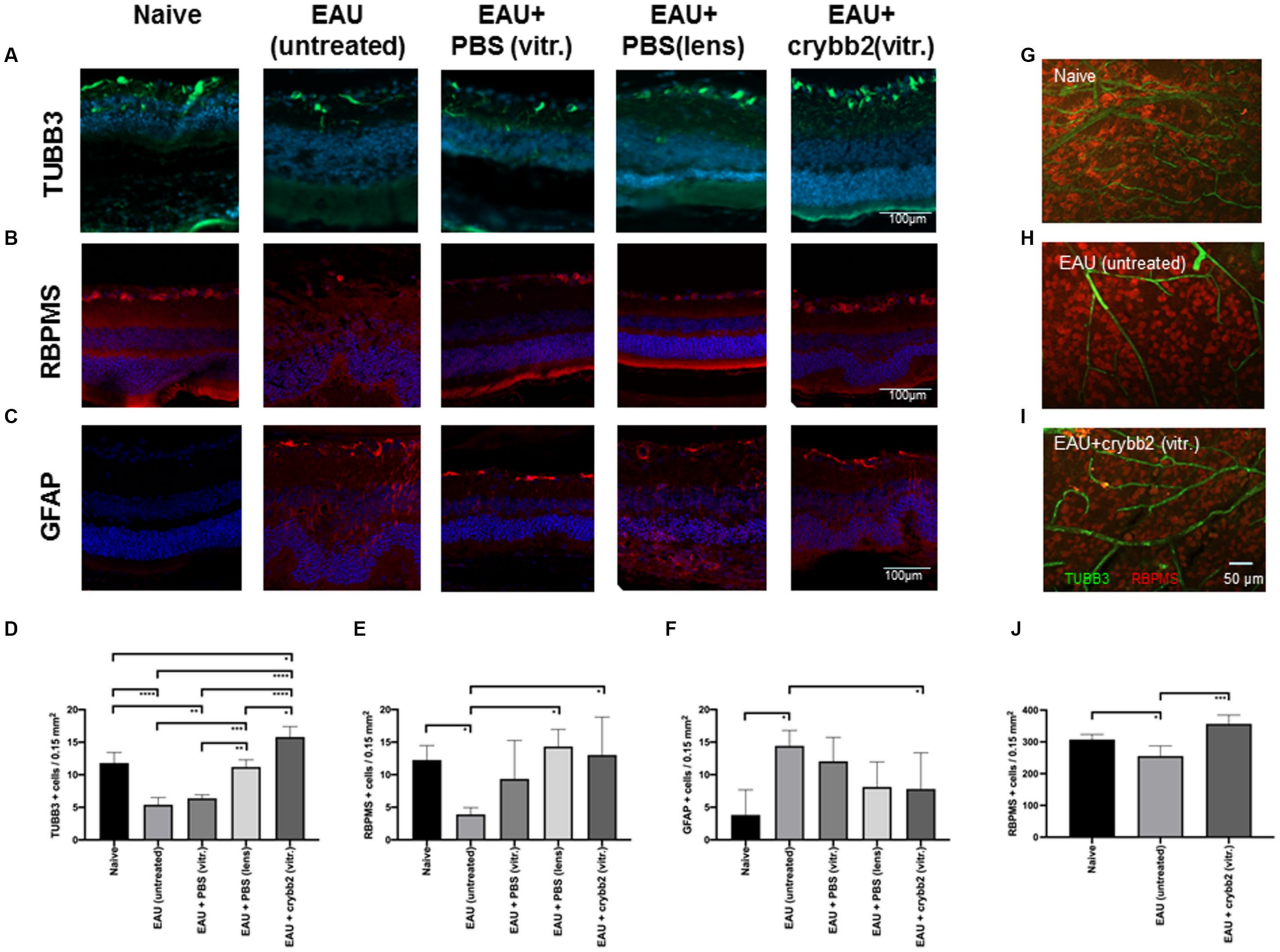
Expression and localization of TUBB3 (green), RBPMS (red) and glial fibrillary acidic protein (GFAP; red) in naïve retinas or in retinas with EAU and prophylactic treatment with 2 μL rcrybb2 (3 μg/2 μL) or PBS as control. Some mice were also treated with a single intralenticular injection of PBS as a positive control. Mice were immunized 3 days later and eyes were collected 21 days after immunization. The expression of retinal **(A,D)** TUBB3 (green), **(B,E)** RBPMS (red) and **(C,F)** GFAP (red) was determined by immunofluorescence staining of sections (7 μm) from mice that were naive, with EAU (untreated), EAU + PBS (vitreous), EAU + PBS (lens), or EAU + rcrybb2 (vitreous) on day 21 p.i. Positive cells were counted in 3 different section per eye (*N* = 5 eyes per group) and data are expressed as mean ± SD. Scale bar: 100 μm. **(G,H,I)** The number of retinal RBPMS+ cells (red) in naïve, EAU (untreated) and EAU(rcrybb2)-treated retinas was determined by flatmount staining (*N* = 4 per group). Scale bar: 50 μm. **(D–F,J)** ANOVA with Tukey’s *post-hoc* test: ∗*p* < 0.05. ∗*p* < 0.05; ∗∗*p* < 0.01; ∗∗∗*p* < 0.001; ∗∗∗∗*p* < 0.0001.

The markers TUBB3 and RBPMS were used to investigate the presence and localization of RGCs in the mouse retina. To observe whether EAU could have an effect on RGCs, we compared naïve B10.RIII mice with EAU mice on day 21 p.i. Our results showed that numbers of TUBB+ and RBPMS+ cells in the area of the ganglion cell layer were significantly lower in B10.RIII mice with EAU than in naïve mice [Figures 2A,B,D,E; TUBB3+: naïve (11.8 ± 1.64) vs. EAU (untreated; 5.4 ± 1.1): *p* < 0.0001; naïve (11.8 ± 1.64) vs. EAU + PBS(vitreous; 6.4 ± 0.54): *p* < 0.01; RBPMS+: naïve (12.25 ± 2.23) vs. EAU (untreated; 3.91 ± 1.03): *p* < 0.05]. These results indicate RGC loss in the B10.RIII EAU mouse model (Table 1).

**Table 1:** Expression of TUBB3+, RBPMS+, and GFAP+ cells in the retina.

Cell type / 0.15mm^2^	Group	Mean	SD
TUBB3+	Naive	11.80	1.60
	EAU (untreated)	5.40	1.10
	EAU + PBS (vitr.)	6.40	0.50
	EAU + PBS (lens)	11.20	1.10
	EAU + rcrybb2 (vitr.)	15.80	1.60
RBPMS+	Naive	12.25	2.20
	EAU (untreated)	3.91	1.00
	EAU + PBS (vitr.)	9.35	5.90
	EAU + PBS (lens)	14.33	2.60
	EAU + rcrybb2 (vitr.)	13.02	5.80
GFAP+	Naive	3.83	3.80
	EAU (untreated)	14.43	2.40
	EAU + PBS (vitr.)	12.07	3.70
	EAU + PBS (lens)	8.13	3.80
	EAU + rcrybb2 (vitr.)	7.80	5.60
RBPMS+(Flatmounts)	Naive	307,40	15,97
	EAU (untreated)	254.00	41.10
	EAU + rcrybb2 (vitr.)	356.75	32.20

EAU eyes intravitreally injected with rcrybb2 and mice with eye lens injury showed an increase in TUBB3+ and RBPMS+ cells in the ganglion cell layer compared to untreated eyes of EAU mice [TUBB3+: EAU + rcrybb2(vitreous) 15.8 ± 1.6 vs. EAU(untreated) 5.4 ± 1.1: *p* < 0.0001; EAU + PBS(lens; 11.2 ± 1.1) vs. EAU(untreated; 5.4 ± 1.1): *p* < 0.001; RBPMS+: EAU + rcrybb2(vitreous) 13.02 ± 5.8 vs. EAU(untreated) 3.9 ± 1.0: *p* < 0.05; EAU + PBS(lens; 14.33.2 ± 2.6) vs. EAU(untreated; 3.9 ± 1.0): *p* < 0.05; Figures 2A,B,D,E; Table 1].

A similar result was observed when EAU eyes intravitreally injected with PBS were used instead of untreated EAU eyes as a control [TUBB3+: EAU + rcrybb2(vitreous; 15.8 ± 1.6) vs. EAU + PBS(vitreous; 6.4 ± 0.45): *p* < 0.0001; EAU + PBS(lens; 11.2 ± 1.1) vs. EAU + PBS(vitreous; 6.4 ± 0.45): *p* < 0.001]. Furthermore, EAU eyes injected with rcrybb2 exhibited a higher number of TUBB+ cells compared to EAU eyes in which PBS was injected into the lens [TUBB3+: EAU + rcrybb2(vitreous; 15.8 ± 1.6) vs. EAU + PBS(lens; 11.2 ± 1.1): *p* < 0.05].

The cell count of TUBB3-positive cells in the EAU + crybb2 group was found to be significantly higher than that observed in the naive group [TUBB3+: EAU + rcrybb2(vitreous; 15.8 ± 1.6) vs. naive (11.8 ± 1.64): *p* < 0.05; Figures 2A,D; Table 1].

This observation suggests that survival of RGCs in the rcrybb2 group and of lens-injured eyes is increased compared with the EAU-untreated control group or the group receiving an intravitreal injection of PBS.

We also analyzed the expression of GFAP, which has been described as a marker for astrocytes and Müller cells (Eng et al., 2000; Sofroniew and Vinters, 2010). In immunofluorescence sections, we observed an increase in GFAP+ cells in the ganglion cell layer in mice with EAU [GFAP+: EAU (14.43 ± 2.4) vs. naive (3.83 ± 3.8): *p* < 0.05]. In mice with an invitreal injection of rcrybb2, there was a reduction in the number of GFAP+ cells compared to untreated EAU eyes [GFAP+: EAU (14.43 ± 2.4) vs. naive (3.83 ± 3.8): *p* < 0.05; Figures 2C,F].

**Figure 3:**
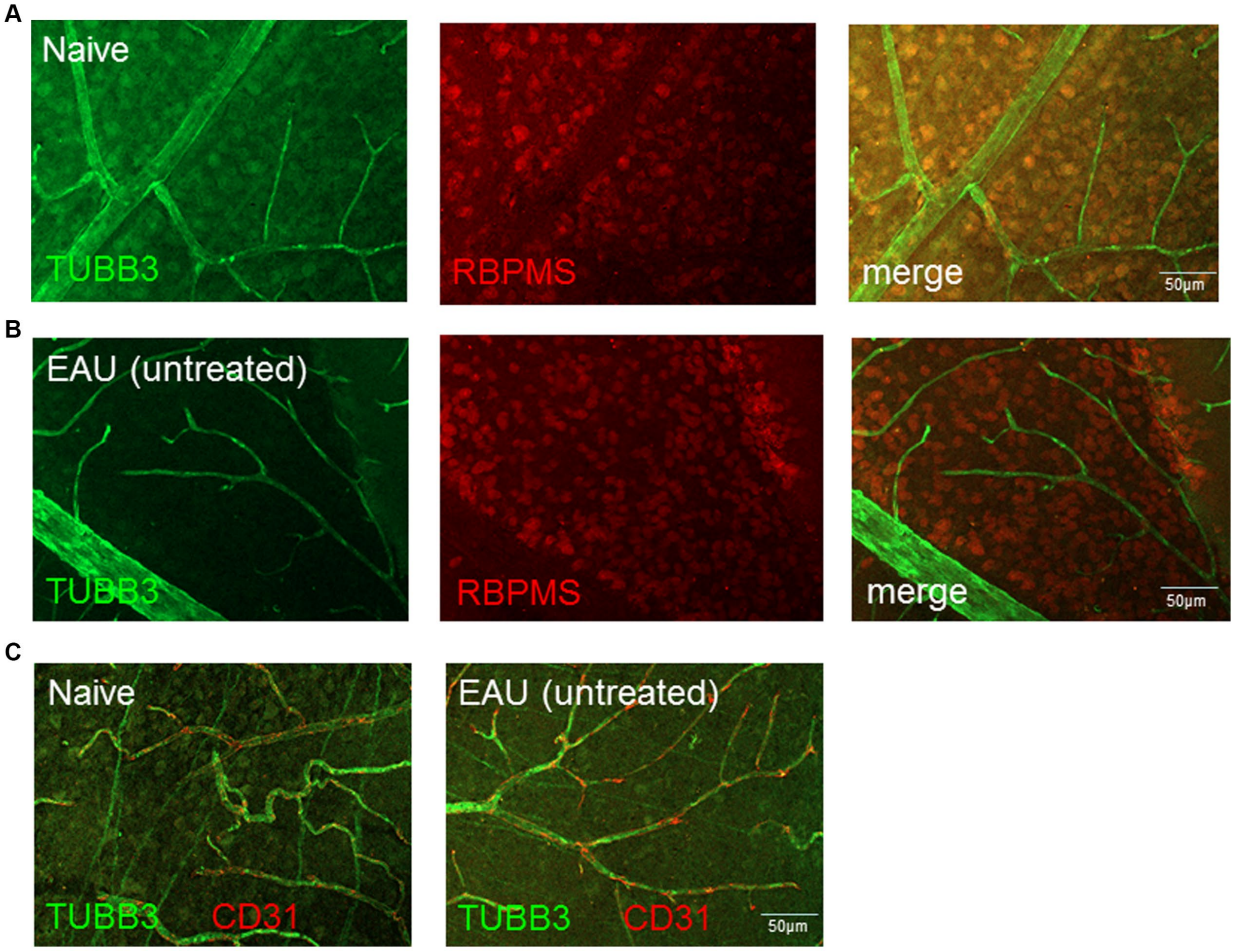
Expression and localization of TUBB3, RBPMS and CD31/PECAM1 in naïve retinas and in retinas with EAU. The expression of TUBB3 (green), RBPMS (red) in **(A)** naïve retinas and **(B)** retinas with EAU was determined by flatmount staining. The results shows that TUBB3 is expressed in retinal ganglion cells (RGCs) but also in **(C)** CD31+ endothelial cells. Scale bar: 50 μm.

The number of RBPMS+ and TUBB3+ retinal ganglion cells in naïve, EAU and EAU + crybb2(vitr.) was analyzed using retinal flat-mount staining. In retinas treated with rcrybb2 or in naïve retinas, the number of RBPMS+ cells was significantly higher than in untreated EAU retinas [RBPMS+: naïve: 307.4 ± 15.97 vs. EAU(untreated): 254.00 ± 41.1: *p* < 0.05; EAU + rcrybb2(vitreous): 356.75 ± 32.2 vs. EAU(untreated) 254.00 ± 41.1: *p* < 0.01] (Figures 2G–J) (Table 1). The TUBB3 staining for retinal ganglion cells in flat-mount retinas was not included in the analysis due to the insufficient staining intensity observed, particularly in the EAU retinas (Figures 2G–I, 3A,B). Conversely, positive staining for TUBB3 was observed in all groups within the CD31+ endothelial cells, indicating that in the mouse retinas, the marker TUBB3 is not exclusively confined to retinal ganglion cells (Figure 3C).

**Figure 4:**
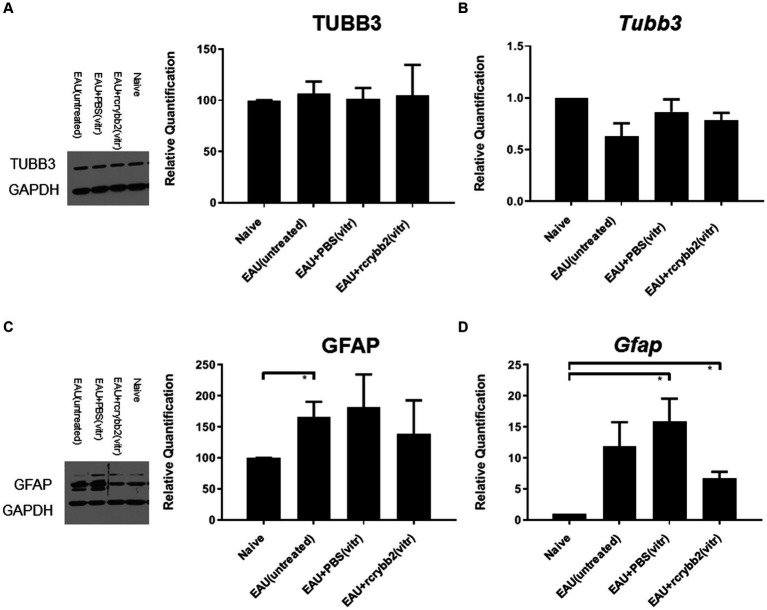
Western blot analysis and RT-qPCR graphs of correlated relative densities. Blots of **(A)** TUBB3 and glyceraldehyde 3-phosphate dehydrogenase (GAPDH) and their correlated relative densities, each with the corresponding control containing GAPDH. **(B)** mRNA level of Tubb3 as measured by RT-qPCR analysis. Blots of **(C)** GFAP and GAPDH and their correlated relative densities, each with the corresponding control with GAPDH. **(D)** mRNA level of Gfap as measured by RT-qPCR analysis. Retinal samples were obtained from naïve eyes, EAU (untreated), EAU + PBS (vitreous), or EAU + rcrybb2 (vitreous). Mean ± SEM. Student’s *t*-test, ∗*p* < 0.05.

We then performed Western blotting (Figures 4A,C) and RT-qPCR (Figures 4B,D) to determine the retinal expression levels of TUBB3 and GFAP in the different experimental groups at 21 days p.i. (Table 2).

**Table 2:** Western Blot and RTqPCR analysis of TUBB3 and GFAP in the retina.

Relative density	WB		RT-qPCR		WB		RT-qPCR	
Group	TUBB3	SEM	Tubb3	SEM	GFAP	SEM	Gfap	SEM
Naive	100.00	0.0	1.000	0.13	100.00	0.00	1.00	0.00
EAU(untreated)	106.37	12.04	0.63	0.12	165.64	24.82	11.81	3.97
EAU + PBS(vitr.)	101.07	11.05	0.86	0.12	181.66	52.77	15.92	3.65
EAU + rcrybb2(vitr.)	105.10	29.53	0.78	0.07	138.99	53.78	6.73	1.06

Relative optical densitometry revealed not significantly changed levels of TUBB3 in the control group with EAU compared to the naïve mouse group not receiving hIRBPp161-180 immunization. After injecting PBS or rcrybb2, the differences also did not reach the level of significance.

A similar picture was found when RT-qPCR was used to semi-quantify the expression levels *Tubb3* (Table 2).

Relative optical density of Western blotting showed increased levels of GFAP in untreated EAU mice compared with retinas from naïve mice [naïve (100.00 ± 0.00) vs. EAU (untreated; 165.64 ± 24.82): (*p* < 0.05); Table 2].

Increased *Gfap* expression was also found after intravitreal PBS or rcrybb2 injection in immunized mice when RT-qPCR was used to semi-quantify expression levels for *Gfap* gene expression [naïve (1.00 ± 0.00) vs. EAU + PBS(vitreous; 15.92 ± 3.65): *p* < 0.05; naïve (1.00 ± 0.00) vs. EAU + rcrybb2(vitreous; 6.73 ± 1.06): *p* < 0.05; Figure 4D; Table 2].

### Expression of BAX and BCL-2 in intravitreally rcrybb2-treated retinas of immunized B10.RIII mice

BAX belongs to the BCL-2 protein family and has been identified as a proapoptotic protein (Oltvai et al., 1993). In contrast, BCL-2 localizes to the outer membrane of the mitochrondria to promote cell survival and inhibit the actions of proapoptotic proteins (Hardwick and Soane, 2013). Previous studies have shown that the ratio of BAX to BCL-2 can be used to assess the balance between apoptosis and cell survival in the retina (Podesta et al., 2000).

**Figure 5:**
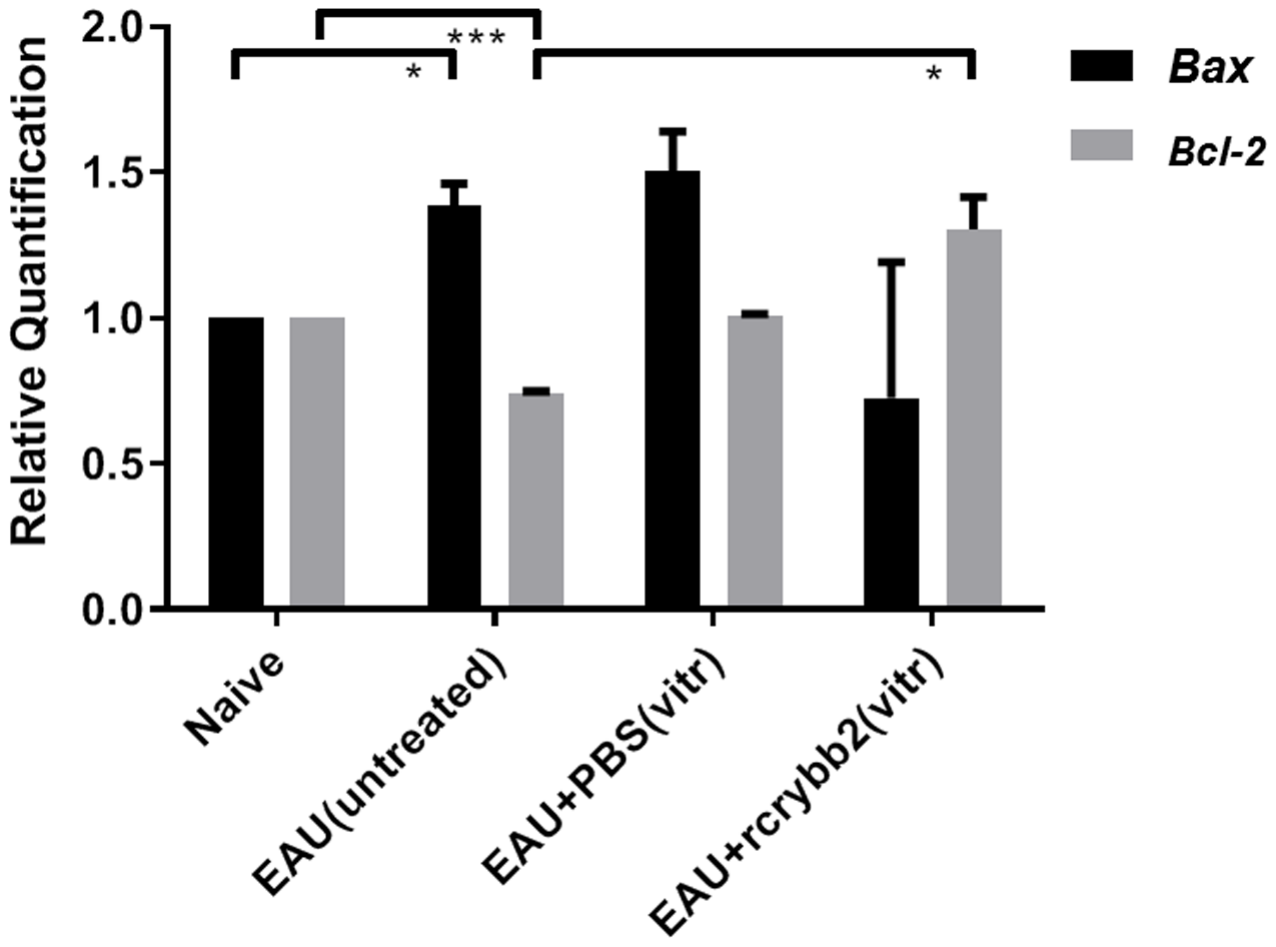
mRNA level of the pro- or anti-apoptotic proteins *Bax* and *Bcl-2* in the retina after treatment with rcrybb2. Gene transcription of *Bax* and *Bcl-2* was measured by RT-qPCR analysis. Retinal samples were isolated from naïve mice, EAU (untreated), EAU + PBS (vitreous), or EAU + rcrybb2 (vitreous). Mean ± SEM. Student’s *t*-test, ∗*p* < 0.05.

Thus, we conducted a comparative RT-qPCR analysis to assess modulation of *Bax/Bcl-2* levels in the retinas of B10.RIII mice with EAU on day 21p.i. as shown in Figure 5. *Bax* mRNA levels were slightly increased in the EAU (untreated) control group compared to the naïve mice [naïve (1.00 ± 0.00) vs. EAU (untreated): *p* < 0.05; Figure 5].

*Bcl-2* mRNA levels were lower in B10.RIII mice with EAU than in naïve mice [naïve (1.00 ± 0.0) vs. EAU (untreated; 0.74 ± 0.01): *p* < 0.005]. After intravitreal injection of rcrybb2, there was a significant increase in *Bcl-2* mRNA compared to the immunized but untreated control group [EAU (untreated; 0.74 ± 0.01) vs. EAU + rcrybb2 (vitreous; 1.3 ± 0.11): *p* < 0.05]. Taken together, these findings indicate that injecting rcrybb2 into the vitreous prior to inducing EAU causes changes in the level of *Bax / Bcl-2*, which helps promote cell survival while inhibiting apoptosis (Table 3).

**Table 3:** mRNA levels of Bax and Bcl-2.

Relative density	RT-qPCR		RT-qPCR	
Group	Bax	SEM	Bcl-2	SEM
Naive	1.00	0.00	1.00	0.00
EAU(untreated)	1.38	0.08	0.74	0.01
EAU + PBS(vitr.)	1.50	0.14	1.01	0.00
EAU + rcrybb2(vitr.)	0.73	0.47	1.31	0.11

### Influence of intravitreally injected rcrybb2 on the incidence and severity of EAU

**Figure 6:**
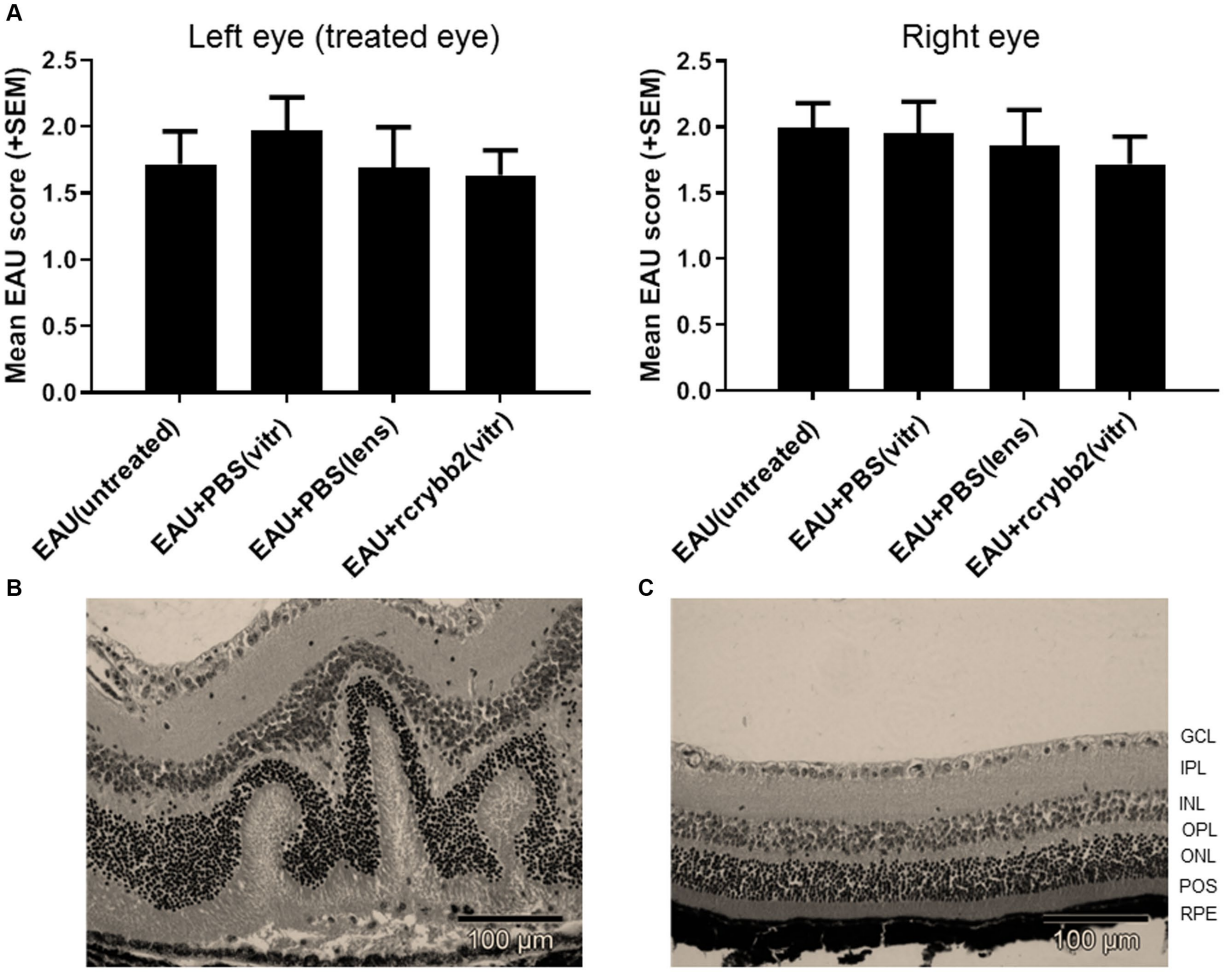
Severity of EAU after intravitreal injection of 2 μL rcrybb2 (3 μg/2 μL) or PBS as control. The lens is a site of crystallin origin; therefore, some mice were treated with a single intralenticular injection of PBS as a positive control. Mice were immunized 3 days later and eyes were collected 21 days after immunization. Statistical differences of EAU scores of the eyes between groups were calculated by using the Kruskal-Wallis test with *post-hoc* analysis. The analysis showed that there was no significant difference between the different treated groups. In addition, the differences between the left (treated) and right (untreated) eyes were not statistically significant as determined by U-test. **(A)** Histologic EAU scores of the different groups: EAU (untreated), EAU + PBS (vitreous), EAU + PBS (lens), EAU + rcrybb2 (vitreous; *N* = 20 each group). **(B)** Representative section of a mouse retina with EAU (clinical score: 2) showing retinal folding. **(C)** Section of a normal mouse retina. Hematoxylin–eosin staining. Scale bar: 100 μm.

We next investigated whether the intravitreal injection of rcrybb2 could influence the course of EAU in immunized mice (Figure 6). The development of clinically monophasic EAU generally begins at day 5–11 p.i. and reaches a maximum of leukocyte infiltration at day 14 p.i. After peak disease, the retina of the eye does not return to its predisease phenotype and shows fluctuations in the number of infiltrating leukocytes and changes to their relative composition (Kerr et al., 2008).

In our study, untreated control mice (*n* = 20) showed moderate EAU scores for retinal detachment, vasculitis, retinitis, vitritis, and photoreceptor cell damage at day 21 (EAU score = 2). Intravitreal treatment with rcrybb2 did not improve EAU scores compared to the untreated control mice. The differences in untreated control mice as compared to those that received a PBS injection into the vitreous or a PBS injection into the lens also did not reach the level of significance in our experiments (Kruskal-Wallis test: n.s.).

The severity of EAU was similar in the contralateral untreated (right) eye in all groups, which also did not reach the level of significance (Kruskal-Wallis test: n.s.). Finally, the severity of EAU in the left (treated) eye was compared with that in the right (untreated) eye. Statistical significance was not reached (Figure 6; Table 4).

**Table 4:** Course of EAU after treatment with rcrybb2.

EAU Score	Left eye		Right eye	
Group	Mean	SEM	Mean	SEM
EAU(untreated)	1.72	0.25	1.99	0.19
EAU + PBS(vitr.)	1.97	0,25	1.95	0.24
EAU + PBS(lens)	1.69	0,30	1.86	0.27
EAU + rcrybb2(vitr.)	1.64	0.19	1.72	0.21

### Influence of intravitreally injected rcrybb2 on the systemic immune response after hIRBPp161-180 immunization

**Figure 7:**
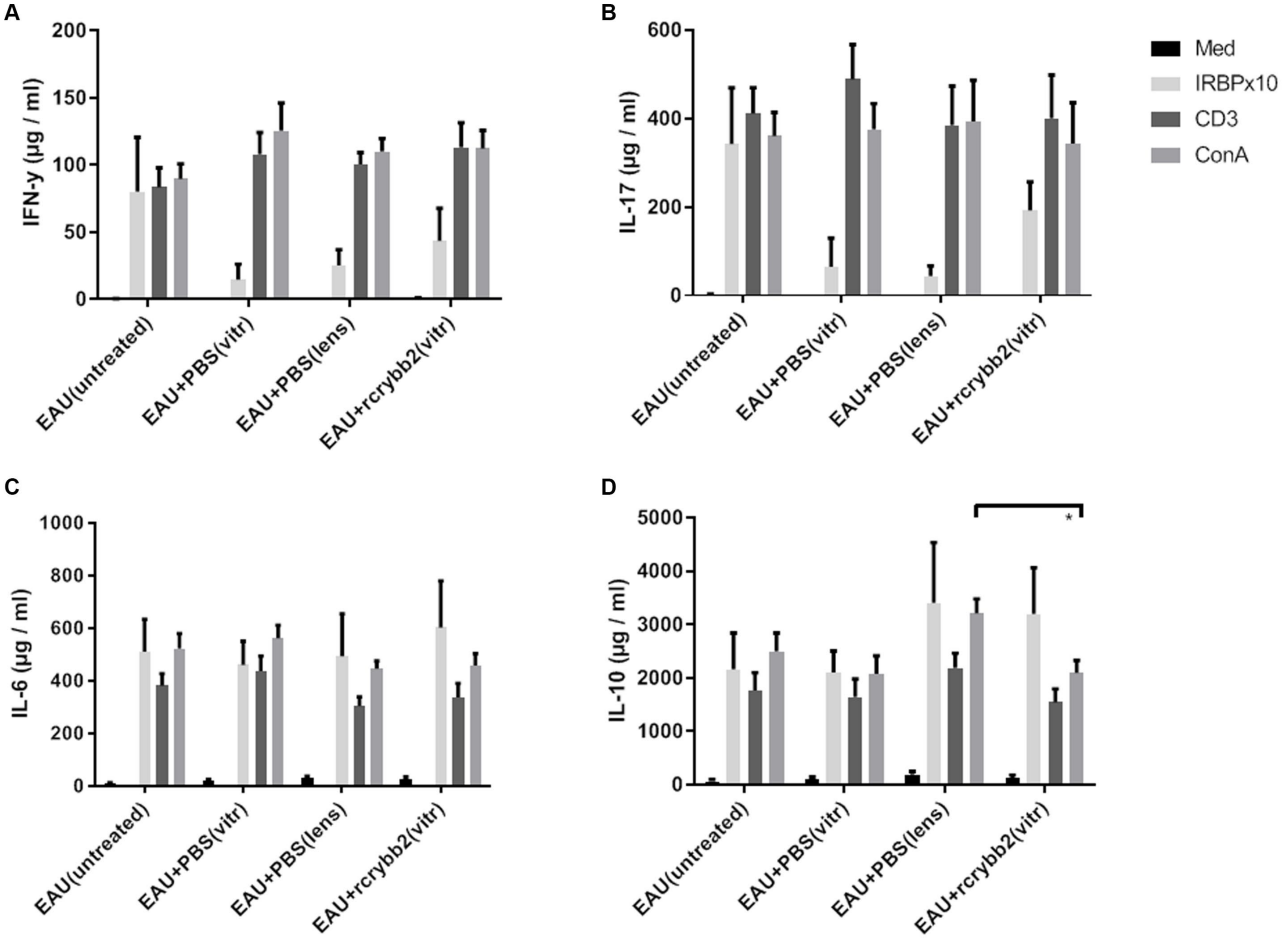
Influence of intravitreal rcrybb2 injection on hIRBPp161-180 specific immune responses. Splenocytes were cocultured with medium, hIRBPp161-180, CD3, or ConA. Splenocyte supernatants from immunized mice were collected 24 h after stimulation and assayed for the indicated cytokines using ELISA kits. Levels of **(A)** IFN-γ, **(B)** IL-17, **(C)** IL-6, and **(D)** IL-10 after stimulation. Data are expressed as mean ± SEM. ANOVA with Tukey’s *post-hoc* test: ∗*p* < 0.05.

Next, we examined cytokine production in splenocytes isolated from mice after intravitreal injection of rcrybb2 (Figure 7; Tables 5A–D). Although hIRBPp161-180-specific stimulation after intravitreal treatment or lens injury somewhat modulated the response as compared with the untreated control group, the changes in the cytokine response did not reach the level of significance, except in one group [IL-10: ConA: EAU + rcrybb2(vitreous; 2091.95 ± 233.45) vs. EAU + PBS(lens; 3206.26 ± 270.04): *p* < 0.05; Table 5D].

**Table 5:** Expression of cytokines in spleen supernatants.

A. Expression of IFN-y in spleen supernatants								
Cytokines	Med		IRBP		CD3		ConA	
Group	Mean	SEM	Mean	SEM	Mean	SEM	Mean	SEM
EAU(untreated)	0.10	0.10	8.01	4.04	83.31	14.44	89.51	11.28
EAU + PBS(vitr.)	0.05	0.05	1.44	1.16	107.98	16.12	125.43	20.41
EAU + PBS(lens)	0.00	0.00	2.51	1.17	100.37	8.70	109.82	9.72
EAU + rcrybb2(vitr.)	0.47	0.30	4.33	2.43	112.85	18.47	112.18	13.43
B. Expression of IL-17 in spleen supernatants								
IL-17	Med		IRBP		CD3		ConA	
Group	Mean	SEM	Mean	SEM	Mean	SEM	Mean	SEM
EAU(untreated)	1.64	1.64	34.29	12.63	411.21	58.75	360.86	52.89
EAU + PBS(vitr.)	0.01	0.01	6.48	6.48	489.07	78.09	375.80	58.19
EAU + PBS(lens)	0.00	0.00	4.36	2.30	384.57	88.72	393.90	92.75
EAU + rcrybb2(vitr.)	0.00	0.00	19.28	6.37	400.07	97.99	343.87	91.70
C. Expression of IL-6 in spleen supernatants								
IL-6	Med		IRBP		CD3		ConA	
Group	Mean	SEM	Mean	SEM	Mean	SEM	Mean	SEM
EAU(untreated)	7.82	3.00	50.91	12.27	380.10	45.40	521.35	57.62
EAU + PBS(vitr.)	18.01	5.87	45.84	9.03	435.18	58.06	560.30	49.93
EAU + PBS(lens)	28.40	7.87	49.20	16.15	303.23	35.03	445.66	27.67
EAU + rcrybb2(vitr.)	24.57	8.01	60.17	17.73	335.92	52.87	456.27	46.16
D. Expression of IL-10 in spleen supernatants								
IL-10	Med		IRBP		CD3		ConA	
Group	Mean	SEM	Mean	SEM	Mean	SEM	Mean	SEM
EAU(untreated)	61.69	36.63	215.89	68.25	1764.23	334.19	2492.73	347.46
EAU + PBS(vitr.)	105.23	40.37	210.04	39.98	1641.09	336.71	2072.34	336.91
EAU + PBS(lens)	186.15	61.13	340.37	113.18	2183.34	275.25	3206.26	270.04
EAU + rcrybb2(vitr.)	132.14	46.33	319.84	86.04	1545.43	249.11	2091.95	233.45

### Beta III-tubulin is detectable in retinal endothelial cells during EAU

**Figure 8:**
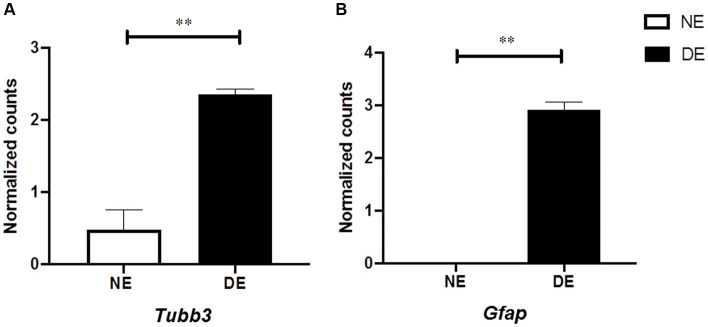
Increased level of *Tubb3* and *Gfap* in retinal endothelial cells in mice with EAU. Single-cell RNA sequencing (scRNAseq) analysis of endothelial cells from eyes with EAU (DE, diseased endothelium, *N* = 3) or healthy control eyes [NE, naïve endothelium, *N* = 4; GSE144168, reported by Lipski et al. (2020)]. **(A,B)** Level of *Tubb3* and *Gfap* calculated using the classical Bayesian algorithm of the “Limma” R package of Network Analyst 3.0. Mean ± SEM, Adj. P: ∗∗*p* < 0.01.

We hypothesized that beta III-tubulin (TUBB3) expression in the retinal endothelial cells of the retina might be increased during the development of EAU, similar to recent findings in a glaucoma model (Prokosch et al., 2020). Therefore, we used scRNA-seq data from murine retinal endothelial samples (four healthy vs. three EAU samples; Lipski et al., 2020). We determined that *Tubb3* level in retinal endothelial cells was significantly upregulated after EAU induction (*Tubb3*, diseased endothelium vs. naïve endothelium, LogFC: 1.8869; AveExpr: 1.2811, *p* = 0.00027912, adjusted *p* = 0.0034113). Similar results were found for *Gfap* (*Gfap*, diseased endothelium vs. naïve endothelium, LogFC: 2.9193; AveExpr: 1.2511: *p* = 1.0866e-07, adjusted *p* = 0.00015471; Figures 8A,B). In conclusion, level of *Tubb3* and *Gfap* was significantly higher in the retinal endothelium after inducing EAU.

## Discussion

Crystallins are dramatically upregulated in numerous pathological conditions of the retina, including mechanical injury, ischemic insult, age-related macular degeneration, diabetic retinopathy, and uveoretinitis (Thanos et al., 2014). Several animal models have shown that crybb2 is significantly upregulated in the regenerating retina. *In vitro,* βb2-crystallin is produced and secreted during axon elongation, while β/γ-crystallins promote axon growth both *in vivo* and *in vitro* by acting either directly by uptake into cells or indirectly by enhancing ciliary neurotrophic factor production from astrocytes to synergistically promote axon regrowth (Liu et al., 2022; Thanos et al., 2014). To date, little is known about the role of crybb2 in the pathogenesis of EAU.

In this study, a single intravitreal injection of rcrybb2 3 days before immunization into naïve B10.RIII mice was used to evaluate the neuroprotective function of rcrybb2 in the development of EAU. It was hypothesized that prophylactic administration of crybb2 would have a greater impact than therapeutic administration, in which EAU is allowed to develop before being treated. We therefore used prophylactic treatment to demonstrate its potential effectiveness. The results show that intracellular fluorescence staining of injected rcrybb2 (as found in the vitreous and in the RGC layer, but not in the deeper retina) remained stable for more than 21 days *in vivo*. Therefore, rcrybb2 was not administered again during our study.

In our experiments, the markers TUBB3 and RBPMS were used to specifically identify RGCs in the ganglion cell layer of the retina in sagittal eye sections (Jiang et al., 2015). Given the uneven distribution of retinal ganglion cells in the retina, sections from three different regions of the eye (*N* = 5) were utilized, with three different regions analyzed (twice in the midperiphery of the retina and once in the center of the eye), ensuring sufficient distance to the optic nerve head (at least 500 μm). The mean values and associated statistical parameters were then calculated from the aforementioned data. The analysis showed that RGCs were significantly reduced in the retinas of mice with EAU compared to naïve mice. Eyes into which rcrybb2 was injected intravitreally showed significantly more TUBB3- and RBPMS-positive cells in the retina than untreated mice with EAU. A similar picture was also found after lens injury.

Astrocytes are specialized glial cells. They are found throughout the entire central nervous system (CNS) and perform essential and complex functions in a healthy CNS. Astrocytes can be detected by a mAb directed against GFAP. Our results showed an increase in GFAP-positive cell after immunization, while a decrease in these cells was observed following intravitreal injection of crybb2. These data suggest that rcrybb2 or lens injury (which was used as a positive control) may protect RGCs from cell death after EAU induction.

The data from the flat mount analysis are in agreement with the results obtained from the mediosagittale histologies. The data demonstrate that eyes with EAU that have been prophylactically treated with rcrybb2 or naïve eyes contain a significantly greater number of RBPMS+ retinal ganglion cells than eyes with untreated EAU. This evidence provides support for the hypothesis that treatment with rcrybb2 protects against degeneration of retinal ganglion cells.

However, the results clearly demonstrate that TUBB3 is not exclusively expressed in RGCs in murine retinas, but rather that CD31+ endothelial cells also express it constitutively.

The data presented in this study are corroborated by single-cell RNA sequencing (scRNA-seq) data from a publication, which investigated retinal endothelia in the experimental autoimmune uveitis (EAU) model in C57BL/6 mice. The data demonstrate the constitutional transcription of *Tubb3* in endothelial cells in naive retinas and an elevated expression in EAU eyes (Lipski et al., 2020).

In a rat glaucoma model, it was demonstrated that while the number of RGCs declined during the progression of glaucoma, the overall level of TUBB3 protein remained elevated. The authors of the study were able to detect the expression of TUBB3 in desmin-, PDGFR-β- and α-SMA-positive pericytes, as well as in endothelin-1-positive endothelial cells in eyes affected by glaucoma (Prokosch et al., 2020).

However, our findings are inconsistent with those of Jiang et al. (2015), who employed a dual staining approach in a glaucoma model in C57BL/6 J mice, utilizing fluorogold and a TUBB3-specific staining, and conducted a comparative analysis between the two staining methods. The findings revealed no significant differences between the two detection techniques, leading to the conclusion that TUBB3 is a reliable marker for retinal ganglion cells (Jiang et al., 2015).

In a separate study, the actual proportion of RGCs was determined by using active tracers (fluorogold or hydroxystilbamidine methanesulfonate), and the results were compared with those obtained using TUBB3 and other markers in a range of species, including mice and rats (Nadal-Nicolas et al., 2023). In rats, the proportion of RGCs recognized by the antibody against TUBB3 exceeded 100% when the active tracer fluorogold was employed. Furthermore, 10% of non-RGCs were also identified. In mice, however, the average proportion of RGCs (with active tracer hydroxystilbamidine methanesulfonate) stained with the antibody against TUBB3 was 83%, with no staining observed in non-RGCs. These data contradict our flat-mount results, showing that endothelial cells express TUBB3.

It is recommended that these disparate outcomes be taken into account in future research employing TUBB3 as a marker for retinal ganglion cells (RGCs).

RGCs are part of the neural retina and are located in the ganglion cell layer, the innermost cellular layer of the neural retina. RGCs receive visual information from photoreceptors via two intermediate neuron types: bipolar cells and retinal amacrine cells. The RGCs relay visual information to the processing centers in the brain, which is transmitted by RGC axons that are bundled together as they exit the posterior part of the eye to form the optic nerve (Nickells, 2012). In addition, a small percentage of RCGs contribute little or nothing to vision, but are themselves photosensitive; their axons form the retinohypothalamic tract and contribute to circardian rhythms and the pupillary reflex, the dilation of the pupil (Maes et al., 2017).

Several diseases are characterized by loss of RGCs and damage to the optic nerve, one of the most common being glaucoma, which causes blindness worldwide (Epstein, 1987; Liu et al., 2022; Osborne et al., 1999; Quigley et al., 1995). Depletion of RGCs in the retina has been observed in several glaucoma models after various insults. These animal models include ischemia/reperfusion injury after short-term occlusion of blood vessels (Osborne et al., 1999), selective vessel ligation (Barnett and Osborne, 1995; Lafuente et al., 2001; Otori et al., 1997), photothrombosis (Mosinger and Olney, 1989), or carotid artery occlusion (Block et al., 1992). Another model, in which the connection between RGC and the brain is eliminated by compressing the axons, also causes specific loss of RGCs (Galindo-Romero et al., 2011; Vidal-Sanz et al., 2017; Villegas-Perez et al., 1993).

It is well known that neuronal cell death in pathological conditions of the retina and brain is often associated with an apoptotic mechanism (Buchi, 1992; Maes et al., 2017; Osborne et al., 1999; Otori et al., 1997). An increase in the number of apoptotic cells has also been found in eyes with glaucoma (Nickells and Zack, 1996). It has been shown that suppression of BAX also reduces RGC loss after nerve crush injury (Goldenberg-Cohen et al., 2011), and measuring the ratio of *Bax/Bcl2* in RGCs may be useful to determine the ratio of apoptosis to survival. Our results showed that inducing EAU by immunization significantly increased *Bax* expression in the retina while the expression of *Bcl-2* was significantly decreased compared with naïve control mice. *Bcl-2* expression in the retina was significantly increased after intravitreal administration of rcrybb2. These results suggest that intravitreal administration of rcrybb2 may alter the homeostasis of EAU-affected retinas to support cell survival and to inhibit cell death of RGCs in the mouse retina.

A previous study reported that systemically administered αA-crystallin, but not αB-crystallin, protected the retina by immunomodulating systemic B- and T-cell immune responses. The authors suggested that the oxidative stress in the mitochondria may originate both in the initial innate immune response as well as via the adaptive immune pathways. Through these immunomodulatory effects, the authors anticipate a decrease in oxidative stress, in apoptosis in the retina, and in photoreceptor degeneration (Rao et al., 2012).

We found that intravitreal rcrybb2 treatment was unable to significantly alter the systemic immune response against hIRBPp160-180 as determined with isolated splenocytes, and the EAU score was not affected. We conclude that prophylactic injection of rcrybb2 may have little or no effect on the systemic cellular immune response and EAU. These results suggest that rcrybb2 may act primarily by reducing apoptosis in RGCs. Thus, rcrybb2 may act downstream of leukocyte activation, e.g., to modulate apoptosis in RGC cells.

There are some limitations in the present study we would like to address.

Although prophylactic treatment with crybb2 did not affect the severity of EAU, our data suggest that it improved RGC survival. An electroretinogram (ERG) could be used as a functional test to demonstrate improvement in visual function. The specific ERG waves affected by RGC loss should depend on the type of RGCs lost. Loss of magnocellular RGCs should primarily affect the b-wave, whereas loss of parvocellular RGCs should primarily affect the oscillatory potentials.

A possible alternative approach would have been to mimic the effect of rcrybb2 *ex vivo* by using primary retinas and astrocytes in conjunction with rcrybb2, as has recently been shown (Liu et al., 2022).

In conclusion, intravitreal administration of rcrybb2 showed a prolonged presence in the ocular tissues. Although the treatment was unable to reduce the incidence and severity of EAU, our results indicate that rcrybb2 was able to protect retinal RGCs from degeneration. This protection seems to be independent of the local or systemic immune responses. Although the clinical relevance of intravitreal administration of rcrybb2 has not been clearly defined yet, it may offer a promising novel therapeutic strategy to avoid RGC loss in particular in degenerative diseases.

## Author’s note

The following previously published datasets were used: Lipski, D.A., Willermain, F. (2020), Retinal endothelial cell phenotypic modification during experimental autoimmune uveitis: a transcriptomic approach, available at: https://www.ncbi.nlm.nih.gov/geo/download/?acc=GSE144168, NCBI Gene Expression Omnibus, GSE144168.

## Data Availability

The raw data supporting the conclusions of this article will be made available by the authors, without undue reservation.

## Footnotes

^1^ https://www.networkanalyst.ca
